# Biomimetic Transferable Surface for a Real Time Control over Wettability and Photoerasable Writing with Water Drop Lens

**DOI:** 10.1038/srep07407

**Published:** 2014-12-10

**Authors:** Ahnaf Usman Zillohu, Ramzy Abdelaziz, Shahin Homaeigohar, Igor Krasnov, Martin Müller, Thomas Strunskus, Mady Elbahri

**Affiliations:** 1 Institute of Polymer Research, Nanochemistry and Nanoengineering, Helmholtz-Zentrum Geesthacht, Max-Planck-Strasse 1, 21502 Geesthacht, Germany; 2 Nanochemistry and Nanoengineering, Faculty of Engineering, Institute for Materials Science, University of Kiel, Kaiserstrasse 2, 24143 Kiel, Germany; 3 Refractories, Ceramics and Building Materials Department, National Research Centre, Dokki, Cairo, Egypt; 4 Institute of Materials Research, Helmholtz-Zentrum Geesthacht, Max-Planck-Strasse 1, 21502 Geesthacht, Germany; 5 Chair for Multicomponent Materials, Faculty of Engineering, Institute for Materials Science, University of Kiel, Kaiserstrasse 2, 24143 Kiel, Germany

## Abstract

We demonstrate a transferable device that can turn wettability of surfaces to sticky or slippy, as per requirement. It is composed of polymeric yarn with a fibrous structure, which can be lifted and placed on any surface to render it the unique wettability properties. We introduce Polyvinylidenefluoride (PVDF) random fiber as biomimetic rose petal surface. When it is decorated with PVDF nanofibers yarns, the random mesh transform from rose petal sticky state into grass leaf slippy state. When it is placed on sticky, hydrophilic metal coin, it converts the surface of the coin to super hydrophobic. Adjustments in the yarn system, like interyarn spacing, can be done in real time to influence its wettability, which is a unique feature. Next, we load the polymer with a photochromic compound for chemical restructuring. It affects the sliding angle of water drop and makes the fibers optically active. We also demonstrate a “water droplets lens” concept that enables erasable writing on photochromic rose petal sticky fibrous surface. The droplet on a highly hydrophobic surface acts as a ball lens to concentrate light onto a hot spot; thereby we demonstrate UV light writing with water lenses and visible light erasing.

Regarding the wetting properties, there has been a growing interest to mimic natural surfaces, as these have perfected for specific purposes over a time of millions of years. So one observes a lotus leaf that is superhydrophobic, i.e. water drop contact angle of more than 150°, on which a water drop can roll randomly for cleaning purposes^1^,^2^. The surface of a grass leaf is also superhydrophobic but is structured in such a way that water drop rolls along the length of the leaf for self-irrigation of the plant^3^. On the other extreme one finds a rose petal leaf to be superhydrophobic but posing so sticky surface to a water drop that it can be tilted upside down without letting the drop go^4^. Such highly specific properties of naturally occurring surfaces are based not only upon their chemical nature but also on a certain physical structure.

For ideal solid surfaces, i.e., one which is perfectly smooth, flat, rigid and which is also chemically homogeneous, insoluble and nonreactive, the wetting characteristics are defined by the Young's equations as a function of the interfacial free energies^5^. However, most of the real surfaces existing in nature or fabricated in laboratories are rough and/or chemically heterogeneous. In such a case, the Young's equation has been modified according to Cassie or Wenzel model^6^,^7^.

In the Cassie state, the drop is not in full contact with the surface and is partly supported on air pockets that are existing between surface features. Thus, it is in a so-called “composite state”. In contrast to this, the Wenzel drop penetrates into the surface feature which results in an increase in contact area. Since the contact line is anchored between the surface features, the surface holds onto the drop and steeper tilt angles are required to initiate its sliding. Similarly, the opposing wetting behaviors of grass leaf and rose petal stem mainly from their surface roughness features^8^,^9^. In general, both of these surfaces have microbumps but these bumps are present in a different arrangement and therefore behave differently towards a water drop. So grass leaf has a Cassie-Baxter surface on which the water drop easily slides but rose petal surface shows a hybrid of Cassie-Baxter/Wenzel surface where droplets could partly penetrate at least the sublevel of the hierarchical roughness by physical mean (i.e. capillary action of microporous micro/nano scale structures) or chemical mean (i.e. hydrophilic interaction) and hence sticks to the petal^10^.

There is a variety of methods available to mimic these structures, which range from templating and molding, plasma etching, electrospinning to acid or solvent treatment and phase separation, besides others^11^,^12^,^13^,^14^,^15^,^16^. These processes restructure the surface in the form of nanotubes^17^,^18^, bumps^19^, fibers^20^,^21^ or spheres^11^ etc., which is as important for special wetting characteristics as is its chemical nature^22^,^23^,^24^. The progress in artificially made natural surfaces has resulted in a plethora of applications, such as self-cleaning coatings on big structures, coatings against snow collection^25^, coatings on boats for low drag^10^, etc. On the other hand, coatings with sticky characteristics can be used as a mechanical hand for microdrop delivery^17^, as an example, but are still in a state of infancy.

Going beyond nature, i.e. acquiring the ability to change wetting properties of a surface in real time would lead to many new applications. Even more, integration of sticky hydrophobic droplet to control the light has potential applications in field of optics but has not been introduced so far.

## Results and Discussion

**Figure 1:**
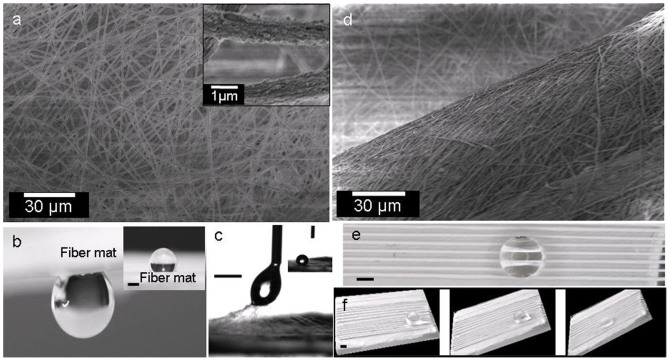
Surface structure and wetting properties. (a) SEM micrograph of PVDF random fiber mat. Inset shows the rough surface of individual fibers. (b) Water drop sticking to a random fiber mat. Inset shows same water drop when right side up. (c) Camera pictures showing fibers being pulled out of random fiber mat, along with the water drop. Inset shows the drop ultimately staying with fibers mat. (d) SEM micrograph showing the surface of half of the yarn. Random fiber mat under the yarn is visible in the background. (e) Top view of a water drop sitting on PVDF yarn that was arranged on PVDF random mesh. (f) Sequence of pictures showing water drop sliding on yarn when it is tilted. Scale bar is 1 mm, unless mentioned.

PVDF was selected as a test polymer in this study. It is a fluoropolymer and consequently shows a very good chemical and moisture resistance. This ability is necessary for its potential application, e.g., in microcapillary fluid delivery, etc, where a polymer may come in contact with different liquids. Moreover, it easily lends itself to electrospinning – the method used for making nanofibers in this study. Electrospinning is a simple and fast method for producing fibers of different composition, surface texture and size. These 1D fibers can be oriented/aligned in 2D to 3D forms either individually or in the form of yarns. Theoretically, these flexible fibers/yarns can be laid on many types of substrate along with their specific wetting properties. The electrospun fibers used for preparing the functional surface had an average fiber diameter of about 1 μm ± 200 nm, Figure 1a, and were collected in the form of a loose mesh. The surface of individual fibers was rough due to presence of nanobumps of size approximately 100 nm or less. PVDF random fiber mat was found to be highly hydrophobic with water drop contact angle of 130 ± 3°, which is much higher compared to that found on its film, i.e., 80°. This increase in contact angle is because of increased surface roughness^26^,^27^,^28^,^29^. Considering a high contact angle, one might expect that a water drop should simply slide on the fibers, but, on contrary, water drop stayed sticking to them and the fibrous surfaces could even be tilted upside down without letting the drop to fall, Figure 1b. Actually, if the drop was pulled away from the fibrous mesh, the fibers would pull out of mesh along the drop Figure 1c. Thus, we have a highly hydrophobic but sticky surface that resembles a rose petal wherein the stickiness came from the porous nature of the electrospun fiber mat^30^, which acts as capillary channels where water could seep into the spaces between the fibers and lock the drop to the surface.

An increase in contact angle of polymer nanofibers compared to that of the bulk film is already known^31^,^32^,^33^. However, turning the same fiber from being sticky to a slippy, has not been introduced so for to best of our knowledge. For many applications, a surface not only needs to be superhydrophobic but also water should not stick to it, i.e., it should show the so-called grass leaf effect.

**Figure 2:**
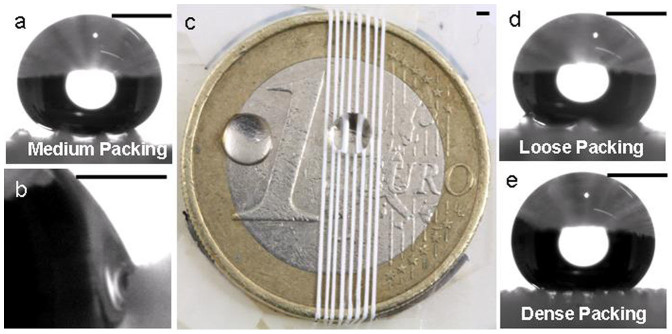
Device application. (a) Water drops sitting on yarns with medium packing. (b) Anchoring of water drop on a yarn. (c) A coin covered with yarns with water drops on the coin and the yarns. (d) Water drops sitting on yarns with loose packing. (e) Water drops sitting on yarns with dense packing. Scale bar, 1 mm.

As mentioned earlier, that a surface can be modified from a being a sticky to a slippy one by several methods, like plasma treatment^34^, electrochemical deposition^35^, or chemical vapor deposition^36^, etc. However, here we convert a rose petal like, hydrophobic sticky character of the surface to a grass leaf like slippy one, by physical rearrangement of surface features. This was carried out by lying on the random mesh, a hierarchical structure composed of yarns prepared with electrospun nanofibers. To prepare a yarn, the fibers were simply twisted and pulled manually in the form of threads of ~250 μm average diameter. Twisting of the fibers into yarn brought the fibers densely close together and they appeared as dense nanostructured ridges on random fiber mats, as seen in the SEM micrograph, Figure 1d. These yarns were then laid on the random fiber mat with interyarn spacing of about 500 μm (called “medium packing” onwards), Figure 1e. The structure thus formed can be called an artificial grass leaf on which the drop could slide at a tilting angle of about 45°, Figure 1f. The sliding of the drop on a yarn like surface roughness could be attributed to the fact that the drop was not completely touching the surface rather it was supported by the yarn only at some points and the rest was suspended in air, Figure 2a. Therefore, it was in a kind of “composite state”. The twisting and rolling carried out during yarn formation also decreased the number of loose fibers on its surface which otherwise could act as pinning sites against the drop sliding. The compaction of structure and a decrease in loose fibers on yarn surface made the surface slippy towards a water drop. This, together with the presence of air pockets under the drop and between the yarns eased the sliding of the drop. Even though the yarn had much lesser pores which otherwise could be the pinning site for the drop as in the case of random mesh, but still these pores were not completely eliminated, Figure 1d. As a result, the sliding started only after breakage of the anchor points and the drop accelerated during its motion on the yarn like features. When a drop was placed slowly on the surface its contact line could come to equilibrium with a course of time after its capillary seepage into the tight pores between the nanofibers composing the yarn and got pinned there, Figure 2b^37^. On a tilt, the drop was attached to these points by so-called microcapillary bridges and its movement was only possible by necking and rupture of these bridges. After detachment, the drop accelerated on the surface, unlike its slow motion on a smooth film, due to reduced dynamic friction. The accelerated motion of a moving drop starts due to additional energy stored in the stretched column, which was released on its breakage. Further pinning of the drop during its travel on the yarn was sometimes observed, but was generally absent due to momentum of the drop so that it could not track the surface inhomogeneities, giving rise to dynamic hydrophobicity^37^.

A big advantage of yarn like setup is that it can be removed from one surface and placed onto another along with its hydrophobic properties. As an example, the same yarn was removed from random fiber mesh and placed on a metal surface (a coin) to convert it into a slippy hydrophobic surface, Figure 2c. It is obvious that the contact angle of the drop with yarn was hydrophobic but with the bare metal surface, it was hydrophilic. On tilting the coin the drop slipped on the yarn at around 42° tilt while it did not leave the bare coin even on an upside down tilt. In fact, our setup can be devised to change the interyarn spacing in real time to affect the sticky/slippy behavior of the surface.

Having proved the switching of the surface from rose petal (random fiber mesh) to grass leaf (yarn on top of random mesh) by surface rearrangement, we further demonstrate the ability of our device to change the sliding angle by simply adjusting the distance between yarn, which can be done in real time by proper arrangements. We placed the yarn on the random mesh with almost no interyarn spacing (called “dense packing”) and with 1 mm spacing (called “loose packing”) which was about double to that used in the “medium packing”. It is to be noted that the yarns used for different packing densities had similar size and only the interyarn spacing was changed. The average tilt angle required to initiate sliding of drop on yarn with different packing density is presented in Table 1. The medium packing arrangement of yarn seemed to be the most efficient as yarn with both “loose packing” and “dense packing” needed steeper tilt angle to initiate the drop slide. A small (~8%) variation in the results was observed which might have arisen from the dynamic nature of the test. Despite that the core concept of switching the sticky/slippy state of the surface just by physical rearrangement of the same fibers into yarns, remains firm.

It can be seen in Figure 2d that in the case of “loose packing” the distance between the yarns was so big that the drop could curve into the space between the yarns and could touch the random fiber mat under the yarn. This not only increased the contact area of the drop with the surface but also brought in the sticking effect offered by the random fiber mat lying under the yarn. That is why; a drop sitting on the “loose packing” required the steepest tilt angle to slide. “Dense packing”, Figure 2e, presented a behavior that was average between “medium packing” and “loose packing”. Here, even though the drop was not in contact with the random fibers mat as in the case of “loose packing” but still the air pockets under the drop, as offered by “medium packing”, were also missing. An increase in the contact area with the surface meant more anchoring sites that hindered the easy sliding of the drop. The contact angle hysteresis (Δθ), i.e. the difference of advancing (θ_a_) and receding (θ_r_) contact angles of a drop on a tilted surface, is generally taken as an indicator of the drop's resistance against sliding, with a pinned drop showing higher values of Δθ. The measured Δθ values for the two extreme cases, i.e., loose packed and dense packed yarn were 67° and 45° respectively. The higher hysteresis value for the loose packed yarn system may be attributed to the pinning of the drop as it curves between the widely spaced yarns and touches the random fiber mat. However, as mentioned earlier that the surface of the yarns still had some pores even after twisting and rolling. These pores were also acting as pinning sites for the drop, Figure 2b. That is why; the hysteresis was high even for the dense packing yarn where the drop was not in a direct contact with the random fiber mat.

**Figure 3:**
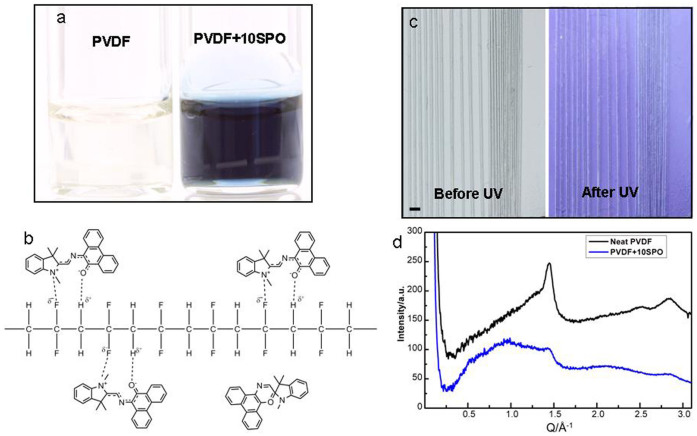
Interaction between SPO and matrix. (a) Blue solution of PVDF-10 wt.% SPO with DMF as a solvent in comparison with colorless solution of neat PVDF. (b) Schematic showing interaction of SPO open form with the PVDF. (c) PVDF yarns with 10 wt.% SPO, before and after UV exposure. Scale bar, 1 mm. (d) WAXS analysis results for neat PVDF fibers and those containing SPO.

The physical rearrangement of the surface, as presented above, is a versatile and easy way to control its wetting properties. On the other hand, the wetting properties of a surface can also be controlled depending upon its chemical restructuring. In short, water being a polar molecule should show stronger adhesion to a polar surface. PVDF can be either polar or non-polar depending upon the conformation of its chains and its crystallinity^38^,^39^. Its crystalline β-phase, which has all the fluorine atoms on one side of the chain, is highly polar^40^. On the other hand, the surface of amorphous PVDF would be less polar because the chains are laid in a random fashion and cancel the effect of each other. So an amorphous structure is expected to pose a less binding surface towards a water drop and its sliding on such a surface is supposedly easier. In contrast to wide wisdom, we believe that even in a highly crystalline polymer, foreign polar molecules can weaken and replace the polymer–polymer interactions with polymer–molecule interactions and hence decrease its crystallinity^41^. Indeed SPO can be switched between “opened, highly polar” and “closed, less polar” form not only on exposure to UV and white light respectively but its open form is also stable in its solvents^42^. Accordingly, 10 wt.% spirooxazine (SPO) was blended with the polymer solution in Dimethylformamide (DMF) that acted not only as a common solvent for polymer and SPO but also caused the SPO molecules to convert into their “open, highly polar” form by creating a polar environment around them. This was evident by the dark blue color of PVDF-10 wt.% SPO solution, Figure 3a. DMF evaporated during electrospinning but in the mean time part of SPO open form was stabilized by a dipole-dipole interaction with the polymer, Figure 3b. That is why; the fibers had a grayish blue color right after electrospinning, Figure 3c. After blending SPO with the polymer, it was expected that the molecules of the photochromic compound could disturb the regular chain arrangement required for a crystalline structure since they acted as side branches and hindered the alignment of chains required for crystallinity, Figure 3b^43^. This could result in fibers being dominantly amorphous with consequent effects on the sliding angle of drop. Indeed, change in crystallinity after adding SPO was seen in the wide-angle X-ray scattering (WAXS) analysis. For the neat PVDF fibers, a strong peak was observed, which suggested that they were dominantly crystalline in either β-phase or its resembling γ-phase, Figure 3d. These two phases are difficult to distinguish from each other by X-ray diffraction, but share a common property that both are polar and thus can ensure good stability of open form of SPO, which is also polar^41^. With the addition of SPO, the strong peak representing crystalline phase was eliminated. As discussed above, the amorphous PVDF being less polar, should offer a surface to the droplet that is only weakly sticky. Thus, the drop could slide on it at a lower tilt angle compared to that in the case of neat crystalline PVDF yarn, Table 2.

However, since a part of SPO embedded in the PVDF fibers was already in the open form, exposure to UV did not bring about such a big change in sliding angle which could be taken as a trend. This was supported by static contact angle measurement, which showed a very small effect, i.e., a change from 135° before UV to 131° after UV exposure.

**Figure 4:**
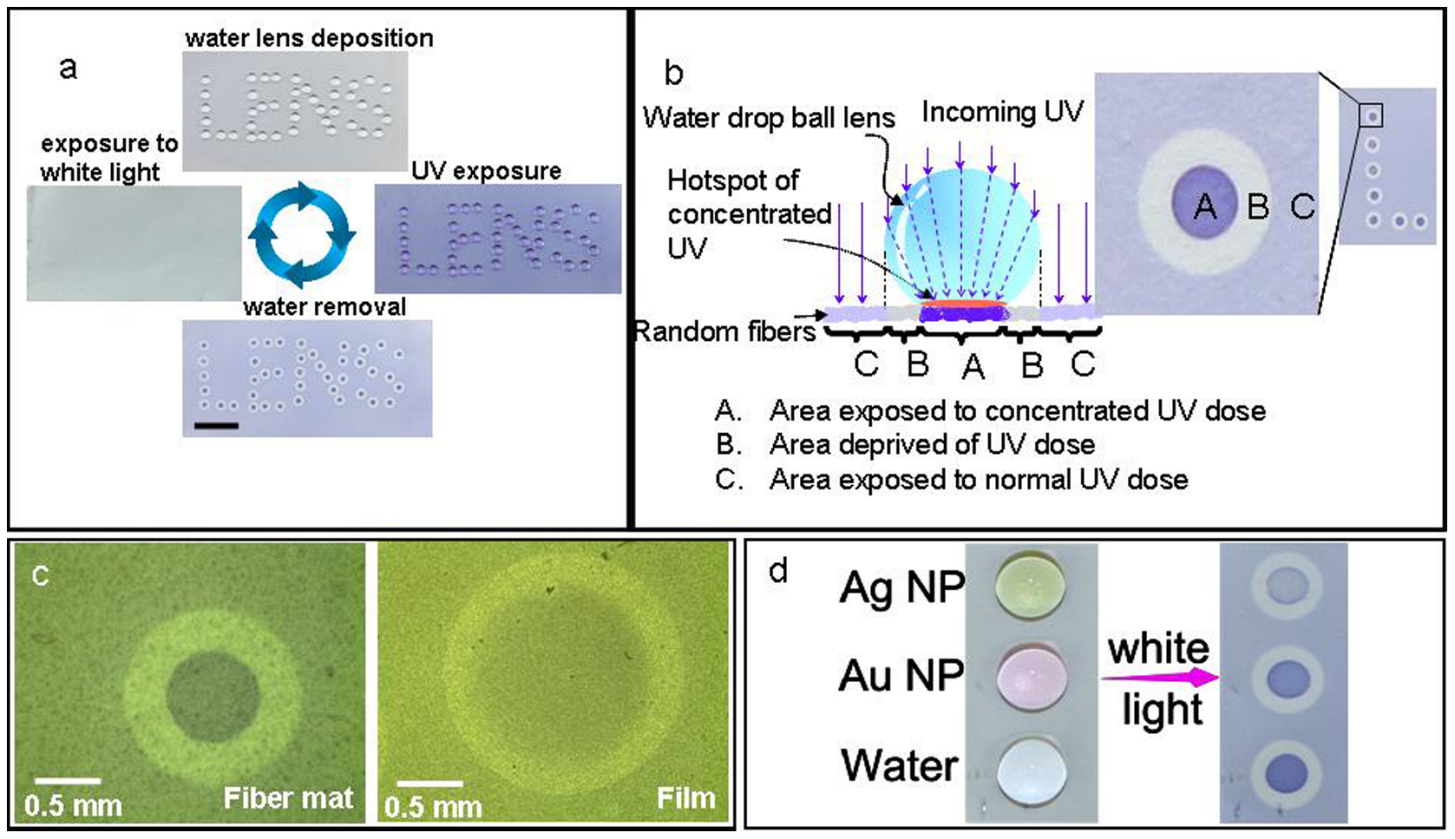
Water drop as a lens. (a) Erasable writing with water lens on ‘PVDF-10 wt.% SPO' random fiber mat. Scale bar, 1 cm. (b) Schematic of the water lens system along with enlarged, top view of one of the spots constituting the letter ‘L'. (c) Optical micrograph of the impression made by water lens on SPO loaded PVDF fibers and film, after UV exposure. (d) PVDF-10 wt.% SPO fibers with water lenses of pure water and that loaded with silver and gold nanoparticles, before (left) and after (right) exposure to white light containing a weak part of UV. Water drop removed after exposure to light in order to show the intensity of the dark impression made by the exposure.

Here it is worth mentioning that only slippy state of a hydrophobic surface is not important but also the sticky state has its own applications. We demonstrate here, the ability of random fibers mat to arrest a drop as a novel way to create two-dimensional patterns by using water drops as tiny ball lenses, Figure 4a. The drops were deposited on a mat of PVDF fibers containing 10 wt.% SPO, to write the word “LENS”. Upon orthogonal UV exposure, water droplets that acted as lenses, deviated the light, concentrating it on the point where the drop was in contact with the fiber mat, thus creating a kind of “UV hotspot”, Figure 4b. Each individual spot consisted of an inner dark blue area, (‘Region A', Figure 4b) because of exposure to highly concentrated UV dose. As the water lens concentrated the light in the centre, the area surrounding the darker region, directly under the curve of the water lens, did not receive any UV exposure and remained in the faint grayish blue color of original fibers (‘Region B', Figure 4b). The rest of the area (‘Region C', Figure 4b) also had a blue color but it was lighter than the ‘Region A' because here, UV was not concentrated. The image formed by selective UV exposure was erasable by intense white light, after which the surface was ready for the next patterning, Figure 4a. On the other hand, a similar pattern could also be made on PVDF film containing 10 wt.% SPO but since the film was not superhydrophobic, therefore the contact area of water drop with the film surface was bigger compared to that in the case of fibers, Figure 4c. Here, the darker area in the middle of impression made by the drop after UV exposure was about four times bigger more than that observed in the case of fibers. This meant that the light concentration effect and the minimum feature size producible on the film were far less than that on the fibers. The advantage of using water is the availability, ease of application and removal and no damage to the delicate substrate, which may be a concern if using other type of lenses.

Enhancement in the field, accompanying as oscillating dipole, due to its proximity with a metal mirror has already been shown^44^. In the present case, water drop acts as a mirror near the oscillating dipoles created by photochromic molecules and a low intensity electromagnetic field of incident UV is intensified (hot spot generation) by multiple reflection/scattering and interference at the interface between the drop and the fibers. Thus, the UV flux can be enhanced on a small area on which potential UV assisted reactions can take place while the rest of the sample surface remains free of any collateral damage. As a potential application of the drop lens, we demonstrate here a local switching using white light that has only a weak part of UV and simultaneously the detection of UV absorbents dispersed in the drop. The intensity of the dark impression made after exposure to light would indicate the nature/concentration of the dispersed species, as shown in the Figure 4d, where water drops containing silver and gold nanoparticles are compared. After exposure to light, the drop that was loaded with gold nanoparticles showed lesser absorption than the drop containing silver nanoparticles. This is because the plasmon of silver (at nearly 400 nm) is very near to the UV range compared to that of gold (which is at nearly 530 nm). This enables one to separate visually, different solution depending upon their UV absorptivity. It can also be seen that UV did not affect the area surrounding the drops as much strongly as it did within the hot spot as seen for the pure water droplet, which actually intensified the UV. The concept of water lens would open a new vein of research ranging from photonic sensors to thermophoresis based on loaded droplets. However, to put these ideas into practice, an in depth investigation of the phenomenon is needed which is beyond the scope of this paper.

## Conclusion

To conclude, it was demonstrated in this work that the wetting properties of a system can be manipulated both by its physical and chemical structure. Sticking of water drop on random mat of polymer nanofibers, and its sliding on the yarn made out of the same nanofibers showed the influence of physical rearrangement. The method of transforming the wetting properties of a surface, as presented here, is simple and unique, with added benefit that this device is removable and can be employed to different types of substrates without causing any permanent change in them. Moreover, it can be switched between the sticky to slippy state instantly. Further control on wetting properties of the system was shown by its chemical modification with a photochromic compound that resulted in crystalline to amorphous transformation of the system with consequent decrease in sliding angle of the water drop. As a novel application of the stickiness of a hydrophobic surface, we used water drop as a lens for writing or making patterns.

## Methods

Polymer solution for electrospinning was prepared by dissolving 11 wt.% of Polyvinylidenefluoride (PVDF) in N,N-Dimethylformamide (DMF). For fibers loaded with 1,3-Dihydro-1,3,3-trimethylspiro[2H-indole-2,3′-[3H]phenanthr[9,10-b](1,4)oxazine] (*i.e.*, SPO), 10 wt.% of it was added to the polymer solution. Electrospinning was done by pushing the polymer solution through a needle that was connected to a high voltage source of 8 kV. Because of electrohydrodynamic nature of the process, the polymer stretched into the form of fibers, which were collected at the base plate in the form of a mat. This mat was simply lifted from the target, spread over a glass plate, and subsequently tested for its wetting properties. Yarns were made by manually rolling and stretching a thin layer of mat collected on the target plate. These yarns were then laid over the glass plate, which was already covered with a layer of random fiber mat. The polymer film was made by blade casting method, from the same solution of PVDF that used for electrospinning of fibers.

Contact angle and sliding angles were measured on OCA15 machine from ‘Dataphysics' using 14 μL water drops. To study the UV effects on contact angle and sliding angle, a UV lamp (Labino AB, 35 W) with its peaks intensity in the range of 365 nm was used. The writing with “water lenses” was carried out by manually delivering of 2 μL water drops on the random fiber mat surface in the form of a pattern. After the first writing, the surface was recovered by exposing it to white LED light source. For analyzing the effect of UV absorbents, suspended in the water, drops of colloidal silver and gold solution containing nanoparticles of size range 5–10 nm were placed over PVDF nanofibers containing 10 wt.% SPO. These were then exposed to a white light (Labino AB, 35 W) for one minute.

For structural characterization, the fibers were glued to a sample frame and consequently exposed to synchrotron X-rays of wavelength 0.65250 Å. The diameter of the beam was about 700 μm. The illumination was done by scans along the direction perpendicular to the fiber axis. The scattered radiation was recorded with a CCD-detector.

For microstructure characterization, the fibers were first sputtered with silver and were consequently examined in Field Emission SEM, Carl Zeiss ULTRA Plus.

## Author Contributions

A.U.Z. performed contact angle, sliding angle and water lens experiments, R.A. did SEM analysis, S.H. performed sliding angle measurements, I.K. performed WAXS analysis, M.M. and T.S. contributed to the manuscript writing. M.E. conceived the idea, designed the experiments, proposed the mechanism, supervised the work and together with A.U.Z. mainly wrote the manuscript.

## Figures and Tables

**Table 1:** Tilt angles required to initiate the drop rolling on surfaces with different roughness. (Drop size, 14 μL)

	Loose packing	Medium packing	Dense packing
Interyarn spacing	1 mm	0.5 mm	Yarns touching each other
Tilt angle for slip	64° ± 5°	45° ± 3°	53° ± 5°

**Table 2:** Tilt angles required to initiate the drop rolling on different surfaces with added SPO. (Drop size, 14 μL)

	Loose packing	Medium packing	Dense packing
Interyarn spacing	1 mm	0.5 mm	Yarns touching each other
Tilt angle for slip	55° ± 2°	43° ± 5°	43° ± 5°
